# Editorial: Plant transcription factors associated with abiotic stress tolerance in crops and wild-relatives

**DOI:** 10.3389/fgene.2024.1431326

**Published:** 2024-07-10

**Authors:** Giuseppe Diego Puglia, Giovanna Frugis, Gitanjali Yadav

**Affiliations:** ^1^ Institute for Agriculture and Forestry Systems in the Mediterranean, National Research Council of Italy, Catania, Italy; ^2^ Institute of Agricultural Biology and Biotechnology, National Research Council of Italy, Rome, Italy; ^3^ Biodiversity Informatics Laboratory, National Institute of Plant Genome Research, New Delhi, India

**Keywords:** drought stress, salinity stress, cold stress, heat stress, hypoxia stress, plant development, phytohormones, transcriptional regulation

Global climate change (GCC), by altering the intensity and frequency of potentially damaging weather events such as droughts, waterlogging, heat waves, and cold spells, has altered seasonal weather patterns, causing severe problems for plant crops and wildlife species (Cramer et al., 2011; Asseng et al., 2015; Minoli et al., 2019). To cope with these challenges, plants have evolved complex regulatory mechanisms that enable them to respond and adapt to changing environmental conditions, while maintaining a balance between optimal growth and stress (Eckardt et al., 2023).

This Research Topic brings together several contributions that highlight the role of transcriptional regulation in plant responses to abiotic stresses and hypothesise its role in stress tolerance. The studies published in this Research Topic deal with well-recognised groups of transcription factors (TFs), but also with new ones whose association with the response to abiotic stresses has been demonstrated by recent molecular advances. This evidence allows us to shed light on the mechanisms by which plants respond to different stresses, with a focus on abiotic stresses such as salt, drought, cold, and waterlogging.

In the first article, Du et al. studied the involvement of WRKY TFs in the cold response of the mangrove, Kandelia obovata, a cold-sensitive plant that is an essential component in the ecosystems of tropical coastal regions. The WRKY TFs are involved in the regulation of expression under abiotic stress conditions such as cold, heat, and drought. Through genome-wide screening and transcriptome analysis, the authors identified 64 KoWRKYs genes, nine of which showed a clear pattern of expression induced by cold stress. This provides a basis for further research into the mechanisms by which this plant responds to cold stress and underlines the importance of conserving this fragile ecosystem.

El Moneim et al. embarked on a journey to discover the evolution and expression dynamics of the Nuclear factor-Y, B subunit (NF-YB) transcription factor in the pepper, Capsicum annuum, under salinity stress. They observed a pronounced response in roots, which aligns with the established knowledge that roots act as primary sensors during salinity stress, regulating both root and shoot adaptation responses. Among the NFYB family genes that have been found, CaNFYB01, CaNFYB18, and CaNFYB19 clearly show transcriptional changes that are linked to the early stages of salinity stress. This suggests that they play a key role in the onset of stress defence mechanisms. This contribution sheds light on the important role of the NFYB TF family in pepper plant physiological responses, especially during environmental stresses.

Plant hormones play a pivotal role in the response to stress, besides regulating important aspects of plant development and growth (Waadt et al., 2022). Three articles highlight the role of transcription factors that act in hormonal signalling to mediate stress responses and plant growth.

The review by Loreti and Perata focuses on the Ethylene Responsive Factors (ERFs) superfamily of transcription factors, particularly on the group VII (ERFVII). ERFVII proteins play an important role in the response to low oxygen conditions, a stress that plants experience in adverse environmental conditions such as sudden floods and severe rainfall that negatively affect plant growth and crop production. The authors discuss how the studies on rice tolerance to flooding helped in understanding the mechanisms of adaptation to hypoxia, leading to the identification of key ERFVII TFs such as SUB1A and to the discovery of an oxygen sensing mechanism in plants. Conservation of ERFVII function in monocots and dicots is also explored, together with the possible application of ERFVII and oxygen sensing research in crop adaptation to flooding. The crosstalk between ethylene, ERFVII TFs, and low oxygen sensing is also discussed beyond abiotic stress tolerance in the light of the existence of hypoxic niches inside the plant, such as the shoot apical meristem, as important determinants of growth and development.

The review by Gentile et al. highlights the role of another subgroup of ERF TFs, the Cytokinin Response Factors (CRFs), as key players in the trade-off between stress response and plant growth. As a side branch of the classical cytokinin (CK) two-component signalling pathway, CRFs regulate CK-auxin hormonal interactions during developmental processes and responses to abiotic stresses. Moreover, CRFs function and expression are responsive to oxidative stress and redox status. In the light of the few available functional studies, mainly conducted in Arabidopsis and to a limited extent, in tomato, the authors discuss the role of CRF TFs in the delicate equilibrium between growth and stress response. In particular, the role of CRFs in maintaining the delicate balance between photosynthetic capacity and the onset of senescence is discussed, as it is of particular importance to obtain crop species resilient to multiple adverse environmental conditions without compromising yield. Greater efforts in the characterization of CRFs in crops, mostly neglected so far, are envisaged.

Auxin is one of the main plant hormones that regulates plant development and response to environmental changes. Marzi et al. focused on TFs that specifically modulate auxin levels in response to salinity, drought, and the presence of heavy metals affecting auxin biosynthesis, transport, or signalling. Beyond Auxin Response Factors (ARFs), the authors described other TFs involved in auxin control, such as those belonging to MADS, NAC, or ETHYLENE-INSENSITIVE (EIN) families, which are activated during salt, drought, or heavy metal stress, respectively. They also highlighted the role of miRNAs in this context and proposed new, potentially active auxin-related TFs that may be important for increasing plant resistance and tolerance to abiotic stresses.

Abiotic stresses trigger the concentration of the calcium ion (Ca^2+^), one of the most important intracellular second messenger molecules involved in many signal transduction pathways in plants (Tuteja and Mahajan 2007). The review by Yang et al. sheds light on the Ca^2+^ signal transduction mechanism and how CBL (calcineurin B-like proteins) and CIPK (CBL-interacting protein kinases) play a part in it. Their interactions enable plants to respond to environmental stress, ion homeostasis, biotic stress, and plant development. The study found several CBL and CIPK genes in plants, such as drought tolerance, salinity stress tolerance, cold stress response, and plant development. As for plant development, CIPKs play a crucial role in pollen tube tip growth, cell division, auxin sensitivity enhancement, root growth, and root meristem development. The study also provides a bibliometric analysis of the growing research on these genes and their potential interaction networks.

Collectively, this Research Topic provides evidence for the importance of transcriptional regulation by TFs in plant responses to abiotic stresses. We hope that this knowledge may be useful in future studies aimed at improving plant resistance and tolerance to abiotic stresses exacerbated by GCC effects.
